# Stroke risk after transient ischemic attack in a Norwegian prospective cohort

**DOI:** 10.1186/s12883-018-1225-y

**Published:** 2019-01-03

**Authors:** Fredrik Ildstad, Hanne Ellekjær, Torgeir Wethal, Stian Lydersen, Janne Kutschera Sund, Hild Fjærtoft, Stephan Schüler, Jens Wilhelm Horn, Geir Bråthen, Ann-Grete Midtsæther, Åse Hagen Morsund, Marja-Liisa Lillebø, Yngve Müller Seljeseth, Bent Indredavik

**Affiliations:** 1 Department of Neuromedicine and Movement Science, Faculty of Medicine and Health Sciences, NTNU - Norwegian University of Science and Technology, P.O.Box 8905, N-7491 Trondheim, Norway; 2 Department of Medicine, Stroke Unit, Trondheim University Hospital, P.O.Box 3250, N-7006 Trondheim, Norway; 3 Department of Cardiology, Trondheim University Hospital, P.O.Box 3250, N-7006 Trondheim, Norway; 4 Regional Center for Child and Youth Mental Health and Child Welfare, NTNU, P.O.Box 8905, N-7491 Trondheim, Norway; 5 Department of Clinical and Molecular Medicine, NTNU, P.O.Box 8905, N-7491 Trondheim, Norway; 6 Department of Medical Quality Registries, Trondheim University Hospital, P.O.Box 3250, N-7006 Trondheim, Norway; 7 Department of Neurology, Namsos Hospital, P.O.Box 453, N-7801 Namsos, Norway; 8 Department of Neurology, Levanger Hospital, P.O.Box 333, N-7601 Levanger, Norway; 9 Department of Neurology, Trondheim University Hospital, P.O.Box 3250, N-7006 Trondheim, Norway; 10 Department of Medicine, Kristiansund Hospital, N-6508 Kristiansund, Norway; 11 Department of Neurology, Molde Hospital, N-6412 Molde, Norway; 12 Department of Medicine, Volda Hospital, P.O.Box 113, N-6103 Volda, Norway; 13 Department of Medicine, Ålesund Hospital, P.O.Box 1600, N-6026 Ålesund, Norway

**Keywords:** TIA (Transient Ischemic Attack), Stroke, ABCD^2^ score, Risk factors, Prognosis

## Abstract

**Background:**

Transient ischemic attack (TIA) is a risk factor of stroke. Modern treatment regimens and changing risk factors in the population justify new estimates of stroke risk after TIA, and evaluation of the recommended ABCD^2^ stroke risk score.

**Methods:**

From October, 2012, to July, 2014, we performed a prospective, multicenter study in Central Norway, enrolling patients with a TIA within the previous 2 weeks. Our aim was to assess stroke risk at 1 week, 3 months and 1 year after TIA, and to determine the predictive value of the dichotomized ABCD^2^ score (0–3 vs 4–7) at each time point. We used data obtained by telephone follow-up and registry data from the Norwegian Stroke Register.

**Results:**

Five hundred and seventy-seven patients with TIA were enrolled of which 85% were examined by a stroke specialist within 24 h after symptom onset. The cumulative incidence of stroke within 1 week, 3 months and 1 year of TIA was 0.9% (95% CI, 0.37–2.0), 3.3% (95% CI, 2.1–5.1) and 5.4% (95% CI, 3.9–7.6), respectively. The accuracy of the ABCD^2^ score provided by *c*-statistics at 7 days, 3 months and 1 year was 0.62 (95% CI, 0.39–0.85), 0.62 (95% CI, 0.51–0.74) and 0.64 (95% CI, 0.54–0.75), respectively.

**Conclusions:**

We found a lower stroke risk after TIA than reported in earlier studies. The ABCD^2^ score did not reliably discriminate between low and high risk patients, suggesting that it may be less useful in populations with a low risk of stroke after TIA.

**Trial registration:**

Unique identifier: NCT02038725 (retrospectively registered, January 16, 2014).

## Background

Stroke is a major cause of disability and death worldwide. Transient ischemic attack (TIA) has the same etiology as stroke, and patients with a TIA have been shown to be at high risk of a subsequent stroke although the stroke risk varies in different studies depending on study population and methodology [1, 2].

Several clinical risk scores have been developed to identify TIA patients with high and low early stroke risk in order to triage the patients in primary and secondary care. The ABCD^2^ score from 2007 has achieved particular prominence [3]. The score is based on clinical information that is easily obtained, consisting of age, blood pressure, type of symptoms, duration of symptoms and presence of diabetes (Table 1). Validations of the ABCD^2^ score have given conflicting results regarding accuracy for both short and long term stroke prediction [4, 5]. However, it remains the most widely used risk score in TIA patients, and several guidelines recommend that patients with a high ABCD^2^ score (4–7), indicating high risk of stroke, should receive specialist assessment within 24 h after the onset of TIA, while for patients with a low score (0–3) specialist assessment within a few days after TIA is considered sufficient [6–8].

**Table 1:** ABCD^2^ score

Characteristics		Score
Age	≥60 years	1
Blood pressure	> 140/90 at presentation	1
Clinical symptoms	Unilateral weakness	2
	Speech disturbance without weakness	1
Duration of symptoms	> 60 min	2
	10–59 min	1
Diabetes	Presence of diabetes	1

Prospective cohort studies on stroke risk after TIA stratified by the ABCD^2^ score, have not been performed in Scandinavia. Since stroke patients in Norway and other Scandinavian countries differ from stroke populations in many other countries by having lower post-stroke mortality [9], it is timely to assess if TIA patients also differ when it comes to stroke risk and survival. Moreover, modern treatment regimens and alteration in risk factors in the population make it necessary to estimate the risk of stroke after TIA and evaluate whether the recommended ABCD^2^ risk score is still useful in identifying TIA patients at the highest stroke risk. The primary aim was to establish a large prospective cohort of TIA patients to find the cumulative stroke risk within 1 week, 3 months and 1 year after TIA. Secondary, we evaluated the predictive value of the dichotomized ABCD^2^ score 0–3/4–7. Additionally, 1 year follow-up data on endarterectomy for symptomatic carotid stenosis, and case fatality, was recorded.

## Methods

### Study design and patient selection

In a prospective, multicenter study, named MIDNOR TIA, TIA patients were consecutively enrolled from October, 2012, to July, 2014. All eight hospitals in the geographical and administrative region of Central Norway recruited patients, of which seven were community hospitals and one a university hospital. Only the university hospital had an out-patient service for acute TIA diagnostics and treatment. TIA patients eligible for enrollment were residents of Central Norway aged 18 to 90 years, they were evaluated by a stroke specialist within 2 weeks of their TIA, and living at home with a modified Rankin Scale of ≤3.

### Data collection and follow-up

Stroke physicians performed inclusion according to eligibility criteria after in-person assessment on the hospital ward, or in a few cases in the outpatient clinic, and then recorded the ABCD^2^ score in standardized paper forms that explicitly listed each component of the score. A standardized diagnostic work-up contained as a minimum a thorough patient history, a physical examination, blood tests, ECG, a brain MRI or CT, and carotid Doppler ultrasound or CT angiography. Trained research nurses appointed at each center prospectively registered detailed baseline data using standardized web-based case report forms. Subsequent stroke (ischemic and hemorrhagic) within 1 week, 3 months and 1 year after the index TIA, was recorded by telephone follow-up at each time point. Additionally, all registered strokes were confirmed by using data from the Norwegian Stroke Register, which is the national quality registry for stroke care established by law. Data from the Norwegian Cardiovascular Disease Registry was used for registering deaths and carotid surgery in the 1 year follow-up period.

### Definitions

TIA was defined as an acute loss of focal cerebral or ocular function lasting less than 24 h according to the diagnostic criteria from the World Health Organization (WHO) [10]. The TIA leading the patient to seek medical help, was defined as the index TIA. The WHO criteria were also used for stroke [11].

The blood pressure measurement used for the ABCD^2^ assignment was the first ever recorded after the onset of the TIA, and in most cases it was recorded in the emergency department. Carotid stenosis was defined as a ≥ 50% narrowing of the symptomatic internal carotid artery on carotid imaging, and the diagnosis of atrial fibrillation was based on at least one confirmative ECG prior to or during the investigation.

### Clinical management

The clinical management followed the current treatment guidelines for TIA [12]. Patients were treated with an antiplatelet agent, mainly aspirin, as soon as possible after the TIA. Hypercholesterolemia, hypertension, atrial fibrillation and diabetes were treated according to current guidelines, supplemented with lifestyle advices. Patients with symptomatic, significant carotid stenosis were in the absence of contraindications treated with endarterectomy. Follow-up of secondary prevention was performed by the patients’ general practitioners.

### Statistical analysis

In a large, previous study [3] of TIA patients (*n* = 4809), an ABCD^2^ score of 0–3 (1628/4809–34%) gave a 1 week stroke risk of < 1% and a score of 4–7 (3181/4809–66%) gave a stroke risk of > 5%. Based on these results, we calculated a requirement of 564 patients in the present study (significance level 0.05 and power 80%).

Kaplan-Meier analysis was used to determine the cumulative incidence of stroke, and the log rank test was used to assess for statistical differences in stroke-free survival between the ABCD^2^ groups. Deaths from other causes than stroke were treated as censoring events. The predictive ability of the ABCD^2^ score was quantified by the areas under the curve (AUC) of a receiver operating characteristics curve (ROC). Confidence intervals (CI) for binomial proportions were calculated using the Wilson score method. We performed Cox proportional hazards regression analysis to calculate hazard ratios (HRs), using the low-risk ABCD^2^ group as the reference category.

Descriptive statistics for continuous variables are given as means with standard deviations (SD), and for categorical variables as frequencies and percentages. Statistical analyses were performed using IBM SPSS Statistics (version 23).

## Results

Originally 591 patients were enrolled, but 7 patients later withdrew their consent. Another 7 patients were excluded, either because symptoms lasted for more than 24 h (*n* = 1), or because the diagnostic work-up excluded the diagnosis of TIA (*n* = 6). Thus, the final study population included 577 patients.

Table 2 summarizes the baseline characteristics, the clinical features and the main investigations of the study population. The mean (SD) age of the patients was 71.5 years (11.0). Four hundred and eighty-nine patients (84.7%) were above 60 years of age and 56.7% were male. A total of 467 subjects (82.5%) experienced their first ever TIA. Four hundred and ninety-one patients (85.4%) were examined by a stroke specialist within 24 h and 525 patients (91%) within 48 h after symptom onset. Only 27 (4.7%) were evaluated at the outpatient clinic, whereas the majority of patients were hospitalized. Median length of hospital stay was 2 days. Speech difficulties, motor weakness and sensory deficits were the most commonly reported symptoms. Forty-eight of 520 (9.2%) patients who had intra- and extracranial imaging performed had a symptomatic carotid stenosis. All patients were examined with brain imaging, either with a CT scan (97.7%) or a diffusion-weighted MRI (DWI-MRI) (62.6%), or both, and all patients were evaluated with either ECG or 24-h Holter ECG, or both.

**Table 2:** Baseline characteristics, clinical features and main investigations of the study population

Variable	n (%)
Age in years, mean ± SD	70.5 ± 11.0
Age > 60 years	489 (84.7)
Male	327 (56.7)
Evaluation within 24 h. of TIA onset	493 (85.4)
Medical history	
Former TIA	101 (17.5)
Former ischemic stroke	87 (15.1)
Former myocardial infarction	67 (11.6)
Diabetes mellitus	66 (11.4)
Hypertension	311 (53.9)^a^
Hypercholesterolemia	216 (37.4)^b^
Current smoker	94 (16.3)
Former smoker	222 (38.5)
Modified Rankin score	
0	282 (48.9)
1	195 (33.8)
2	79 (13.7)
3	21 (3.6)
Clinical features	
Speech disturbances	277 (48)
Hemiparesis of arm	193 (33.4)
Hemisensory loss	134 (23.2)
Hemiparesis of leg	115 (19.9)
Hemiparesis of face	115 (19.9)
Hemianopsia	36 (6.2)
Amaurosis fugax	21 (3.6)
Diplopia	19 (3.3)
Investigations	
Brain CT	564 (97.7)
Acute infarction	13/564 (2.3)
Brain DWI-MRI	361 (62.6)
Acute infarction	97/361 (26.9)
Extracranial imaging	520 (90.1)
Significant stenosis or occlusion	48/520 (9.2)
ECG and/or 24-h Holter ECG	577 (100)
Newly diagnosed and known	
atrial fibrillation and flutter	79/577 (13.7)
Medication	At baseline At discharge
Aspirin	162 (28.1) 179 (31.0)
Other antiplatelet agent	12 (2.1) 36 (6.2)
Aspirin + other antiplatelet agent	59 (10.2) 284 (49.2)
Anticoagulation	56 (9.7) 91 (15.8)
Blood-pressure lowering agent	311 (53.9) 356 (61.7)
Lipid-lowering agent	216 (37.4) 483 (83.7)

^a^Using blood pressure-lowering medication

^b^Using lipid-lowering medication

### Stroke risk and case fatality

Five patients had a stroke within 1 week, 19 patients within 3 months, and 31 patients within 1 year, corresponding to a cumulative incidence of stroke of 0.9, 3.3 and 5.4%, respectively. Twenty-seven (87.1%) of the 31 recurring strokes within 1 year were ischemic strokes, and 4 were intracranial hemorrhages.

All strokes within 1 week occurred within the first 2 days after the TIA. The 5 patients experiencing a stroke within 1 week had ABCD^2^ scores of 3, 4, 4, 6 and 6, respectively (mean score 4.6). One of them had atrial fibrillation and one had a symptomatic carotid stenosis, both of these had ABCD^2^ score of 6. Of all included patients, 9.6% versus 10.2% had carotid stenosis and 11.7% versus 14.8% had atrial fibrillation in the low and high risk group, respectively.

In all, 26 of 48 patients with significant, symptomatic carotid stenosis underwent carotid endarterectomy. During the entire follow-up period of 1 year 10 (1.7%) of the patients died and three of them by hemorrhagic strokes.

### ABCD^2^ score and stroke risk

**Fig. 1:**
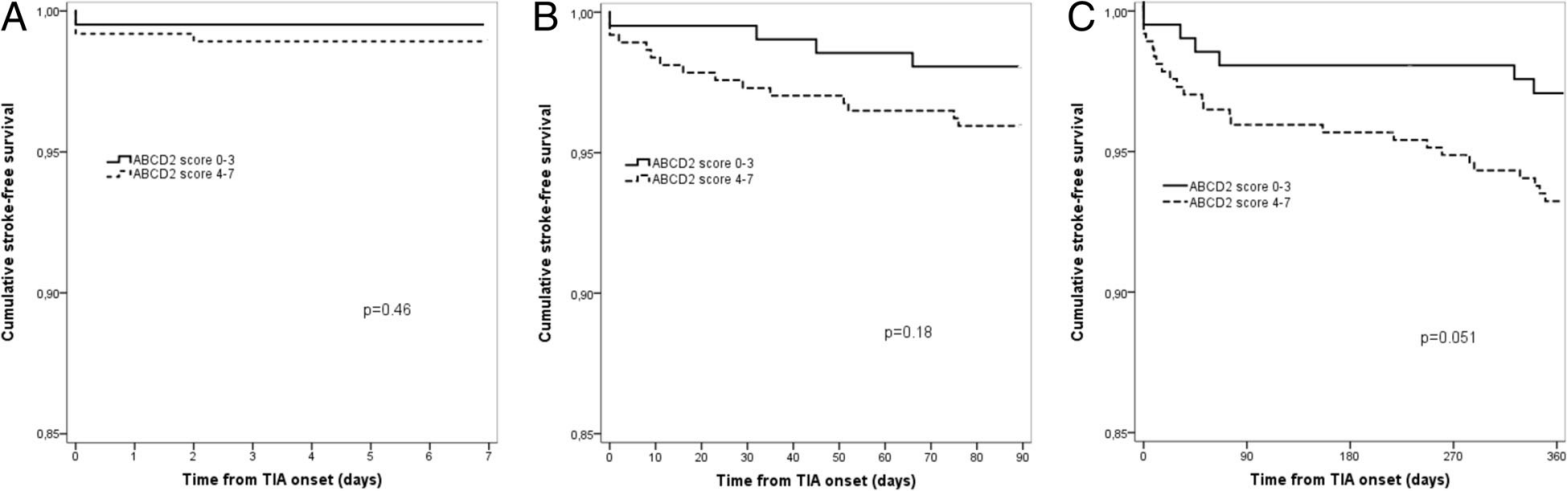
Kaplan-Meier plots of patients surviving free from stroke from time of presenting TIA within 1 week (**a**), 3 months (**b**) and 1 year (**c**) stratified according to ABCD^2^ score 0–3 and 4–7. Log rank tests for differences between the groups

In all, 64.3% (*n* = 371) had a high risk ABCD^2^ score 4–7. The median ABCD^2^ score was 4 (IQR 3–5). Figure 1 shows the Kaplan-Meier curves of patients surviving free from stroke from the time of presenting TIA within 1 week, 3 months and 1 year, stratified according to ABCD^2^ score 0–3 and 4–7. The low risk group shows a higher probability of stroke free survival than the high risk group, although the difference is not statistically significant (*p* = 0.46 at 1 week, *p* = 0.18 at 3 months, *p* = 0.051 at 1 year, log rank test).

The distribution of the ABCD^2^ score with the corresponding stroke rates at each time point is shown in Table 3. In patients with ABCD^2^ score 0 or 1 no strokes occurred at any time point, and for score 2–3 only one stroke within a week. However 19.4% (*n* = 6) of all strokes for the whole period occurred in patients with ABCD^2^ score 2–3. The risk of stroke tended to increase with a higher ABCD^2^ score, with the risk at 1 year ranging from 0% (score of 0 and 1) to 13.2 and 10.5% (score of 6 and 7, respectively).

**Table 3:** The 1 week, 3 months and 1 Year Risks of Stroke According to Each Stratum of the ABCD^2^ Score and Dichotomized Score, with Corresponding AUC Levels for each Time Point

ABCD^2^ score	Patients, n (%)	Stroke events (% of patients)		
		< 1 week	< 3 months	< 1 year
0	7 (1.2)	0	0	0
1	15 (2.6)	0	0	0
2	62 (10.8)	0	0	1 (1.6)
3	122 (21.1)	1 (0.8)	4 (3.3)	5 (4.1)
4	177 (30.7)	2 (1.1)	6 (3.4)	10 (5.6)
5	107 (18.5)	0	3 (2.8)	4 (3.7)
6	68 (11.8)	2 (2.9)	6 (8.8)	9 (13.2)
7	19 (3.3)	0	0	2 (10.5)
< 4	206 (35.7)	1 (0.5)	4 (1.9)	6 (2.9)
≥4	371 (64.3)	4 (1.1)	15 (4.0)	25 (6.7)
Total	577 (100)	5 (0.9)	19 (3.3)	31 (5.4)
AUC^a^ (95% CI)		0.62 (0.39–0.85)	0.62 (0.51–0.74)	0.64 (0.54–0.75)

^a^AUC = Area Under the Curve

**Fig. 2:**
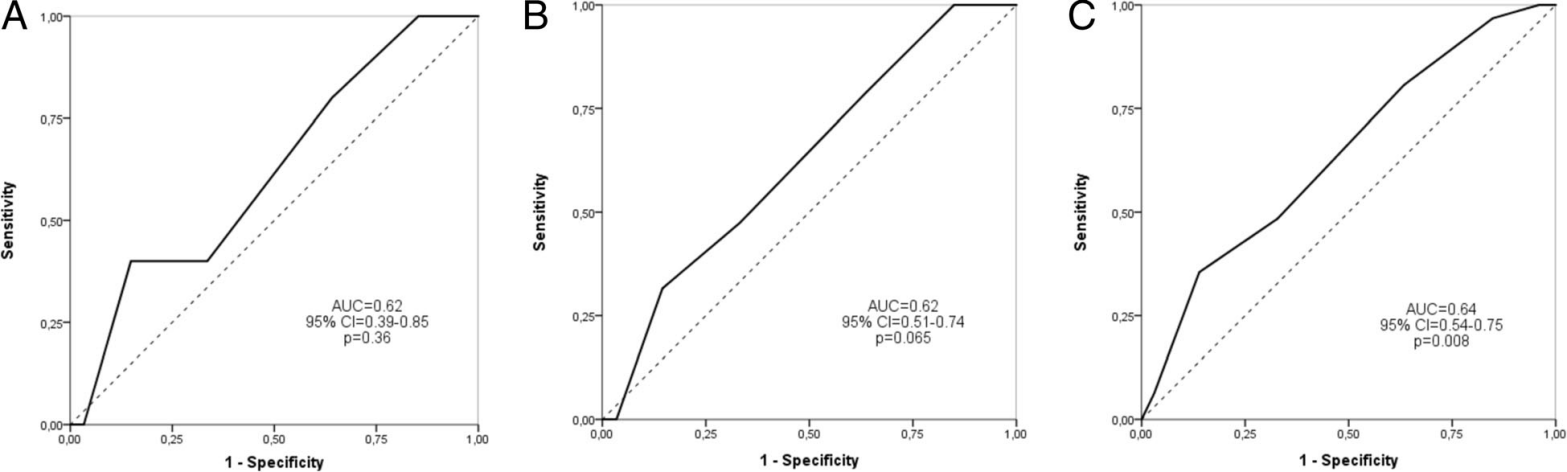
Receiver operating characteristics curves (ROC) for predictive value of ABCD^2^ score within 1 week (**a**), 3 months (**b**) and 1 year (**c**). AUC = Area Under the Curve)

The area under the ROC curve was 0.62 (95% CI = 0.39 to 0.85, *p* = 0.36) at 1 week, 0.62 (95% CI = 0.51 to 0.74, *p* = 0.065) at 3 months, and 0.64 (95% CI = 0.54 to 0.75, *p* = 0,008) at 1 year (Fig. 2). A cox regression analysis comparing high ABCD^2^ score (4–7) with low (reference) score (0–3) showed hazard ratios of 2.22 (95% CI, 0.25 to 19.88, *p* = 0.48), 2.11 (95% CI, 0.7 to 6.35, *p* = 0.19) and 2.37 (95% CI, 0.97 to 5.77, *p* = 0.058) at 1 week, 3 months and 1 year, respectively.

## Discussion

### Stroke risk

We found a low stroke risk after TIA in our study. Both early and late stroke risks were lower than reported in cohorts used to develop and validate the ABCD^2^ score [3], and in several previous TIA cohorts. The pooled stroke risk at 7 days in a meta-analysis published in 2007 reporting from 17 TIA studies performed between 1981 and 2007 was 5.2% [1], 5-fold the risk we found in our study. In the Oxfordshire study the 1 year stroke risk was 11.6% [13], more than two times the stroke risk we found within 1 year. However, the estimated stroke risks in our cohort are in line with the findings in more recent studies [2, 14], including studies evaluating the effect of rapid assessment and initiation of preventive treatment for TIA patients [15–17]. This trend towards a lower stroke recurrence probably reflects both a more rapid evaluation by stroke specialists and improved treatment and secondary prevention strategies implemented for TIA patients during the recent years. In Scandinavia these findings parallel the improved outcome for stroke patients [9], reflecting high quality of initial assessment, treatment and follow-up of both stroke and TIA patients. Differences in socioeconomic status, health economics and health care organization between countries might have an influence on the varying stroke risks found in TIA studies. Performing large TIA studies in different countries provides valuable information on the current post-TIA stroke risk.

To what extent the high hospitalization rate in our study contributed to the low stroke risk is unclear. The aim of the present study was not to compare out-patient and in-patient TIA services. However, in the rapid assessment studies, the EXPRESS study [15] and the SOS-TIA study [16], in which patients were assessed and treated in dedicated out-patient TIA clinics, the very low subsequent stroke rates were attributed to the systematized rapid assessment and treatment initiation. Similarly, in our study, the vast majority of patients, regardless of belonging to low risk or high risk group, were evaluated by a specialist shortly after the event (9 of 10 within 24 h). Only 2% of the patients were enrolled between 1 and 2 weeks after the event. In the small number of patients who were enrolled after 24–48 h from symptom onset, there was one stroke. Excluding these patients from the calculations changes the stroke risks only minimally.

Furthermore, a meta-analysis of 12 randomized trials of aspirin versus control in secondary prevention after TIA or ischemic stroke, identified early administration of aspirin as a key intervention [18]. This may be the main contributor to the low event rate of ischemic stroke during the first days after TIA. In the TIA studies of the meta-analysis from 2007 [1], treatment with aspirin varied considerably, ranging between 47 and 90%. In our study 80.2% of the patients were treated with aspirin, either alone (31%), in combination with dipyridamole (43.7%) or in combination with clopidogrel (5.5%). Aspirin is a simple and low-cost treatment that can be initiated urgently after a TIA, independent of the organization of TIA management on an out-patient or in-patient basis. In contrast, the beneficial effect of other initiated treatments, like antihypertensive and lipid-lowering medication, occurs over time. Promising results regarding dual antiplatelet therapy with aspirin and clopidogrel have been found in two recent studies on stroke risk after TIA or minor stroke [19, 20]. The results however were presented after the initiation of our study, and their clinical implementation need to be validated further.

We acknowledge that neurological symptoms in some enrolled patients might have been caused by non-ischemic conditions, causing a weakening of the association between TIA and stroke risk. However, low risk of stroke explained by misclassification is not likely due to inclusion performed by trained stroke physicians with several years of experience with TIA and stroke patients. Secondly, the ABCD^2^ distribution in our study, with about two thirds of the patients having a high risk score of 4 or more, and a median score of 4, was not towards a lower risk than TIA populations in previous cohorts [3–5]. Thirdly, reclassification of DWI-MRI positive TIAs as stroke can potentially reduce the incidence of subsequent stroke in TIA prognostic studies, since the DWI negative TIAs have been shown to have a lower stroke risk than DWI positive TIAs [21]. All physicians involved in study inclusion were informed to use the time-based TIA-definition. Finally, the mean time from onset to hospital admission was only 17 h, which indicates an appropriate follow-up from TIA onset for most patients and thus prevented loss of stroke-events during the first few days when the risk of stroke after TIA is regarded as high [22].

### ABCD^2^ score

In the low risk ABCD^2^ group there were very few strokes, so a low ABCD^2^ score still indicates a very low stroke risk. However a new and interesting finding was that patients with a high ABCD^2^ score also had a low risk of stroke. Although there were approximately twice as many strokes in the high versus the low risk group at each time point, we did not find significant differences in the Kaplan-Meier analysis. The hazard ratios of 2.1 to 2.4 confirm the same trend towards higher stroke occurrence in the high risk group, but again these were non-significant differences. Furthermore ROC analyses showed insufficient discriminating value of the ABCD^2^ score both when applied 1 week, 3 months and 1 year after stroke.

Only 1 of 206 patients with an ABCD^2^ score of ≤3 experienced a stroke within 1 week. However, as shown in earlier studies, patients with low ABCD^2^ score may have underlying severe pathology, like atrial fibrillation and internal carotid stenosis, which underscores benefit from rapid diagnostic evaluation regardless of risk score [16, 23]. In the present study there were no significant differences in the prevalence of carotid stenosis and atrial fibrillation in the low- and high risk group.

### Strengths and limitations

The strength of the study is the prospective design with the use of standardized diagnostic criteria. The study was conducted in a well-defined geographical region in close collaboration with all the local hospitals and primary health care system. The high adherence to current guidelines regarding assessment and treatment make it a “real-life” clinical scenario, meaning that these findings can be generalized and applied in a broader health-care setting.

The main limitation of our study is the lack of power caused by the low rate of strokes. With a larger cohort we might have been able to show significant differences between the two risk groups. The power calculation was, however, based on current knowledge of post-TIA stroke risk and cannot be considered a methodological error. The fact that our study is not population-based can imply selection bias, as for instance some very mild or short lasting TIAs might not have come to medical attention, or were treated by the general practitioner without referral to the hospital. It is, however, likely that the majority of these patients constitute a low risk group and would have resulted in an even weaker association between TIA and subsequent stroke if included in the analyses. As in most studies, missing data are unavoidable, but the outcome variables were confirmed by using well-functioning national quality registries, and there were no missing ABCD^2^ scores.

## Conclusions

In our study TIA patients had a very low risk of stroke, indicating that the health services in our region offer TIA patients management of high quality. Urgent assessment and intervention are likely the main reasons for the low stroke risk.

Low ABCD^2^ score predicted very low risk of stroke. However, patients with a high score also had a low risk of stroke. Due to the low numbers of stroke, the study did not have sufficient power to detect significant differences in stroke risk between patients with high and low ABCD^2^ score. Our results can still indicate that the ABCD^2^ score may be less applicable to discriminate between high and low stroke risk groups in populations with a low risk of stroke after TIA. Patients with a low score also can have severe underlying pathology, hence rapid evaluation seems to be the key factor for optimizing the outcome in all TIA patients.

## Abbreviations

AUC: Areas under the curve
CI: Confidence intervals
DWI: Diffusion-weighted imaging
HR: Hazard ratios
IQR: Interquartile range
ROC: Receiver operating characteristics curve
TIA: Transient ischemic attack
